# Comparison of the Laryngoscopic View using Macintosh and Miller Blades in Children Less than Four Years Old

**DOI:** 10.25122/jml-2020-0039

**Published:** 2020

**Authors:** Alireza Ebrahim Soltani, Anahid Maleki, Ebrahim Espahbodi, Mehrdad Goudarzi, Parastou Ariana, Alireza Takzare

**Affiliations:** Anaesthesiology Department, Children’s Hospital Medical Centre, Tehran University of Medical Sciences, Tehran, Iran

**Keywords:** Laryngoscopes, Miller blade, Macintosh blade, children, surgery

## Abstract

This study aimed to compare Miller and Macintosh laryngoscopes in zero to 4-year-old children. A total of 72 children with a score of I and II, according to the American Society of Anesthesiologists (ASA) physical status classification, who were candidates for elective surgery with general anesthesia and tracheal intubation were enrolled in the study. The children were divided into two equal groups (36 persons) according to used laryngoscope: Miller laryngoscope (group 1) and Macintosh laryngoscope (group 2). Observations and all laryngoscopies were performed by a single experienced anesthesiologist. Heart rate, systolic blood pressure, non-invasive arterial blood pressure, and hemoglobin saturation were measured and recorded. The number of endotracheal intubation attempts and complications were also recorded for both groups. In terms of gender, the first group consisted of 88.9% boys and 11.1% girls, and the second group consisted of 66.6% boys and 33.3% girls (p-value=0.05). The mean age was 16.7 months in the first group and 17.7 months in the second group (p-value=0.5). The mean weight of the children was 16988.5 g and 16300 g in the Miller and Macintosh groups, respectively (p-value=0.9). Regarding the Cormack-Lehane classification system, 5 patients were classified as grade 1 (13.9%), 14 patients as grade 2 (38.9%), 15 patients as grade 3 (41.7%), and 2 patients as grade 4 (5.6%) in the Macintosh group. In contrast, in the Miller group, 5 patients were classified as grade 1 (13.9%), 27 patients as grade 2 (75%), and 4 patients as grade 3 (11.1%) (p-value=0.004). These results can provide more data about the tracheal intubation method with the Macintosh and Miller laryngoscopes, the ease of intubation, and the best laryngoscopic view with each blade.

## Introduction

Proper airway management by laryngoscopy and tracheal intubation is essential [1]. Laryngoscopy and endotracheal intubation can cause pain and severe sympathoadrenal response, which can result in increased plasma concentrations of catecholamines, blood pressure, heart rate, intracranial pressure, and intraocular pressure, as well as impaired heart rhythm. These changes usually appear within seconds, while sinus tachycardia peaks within two minutes and lasts for five minutes. These variations may be well tolerated in young people without underlying diseases, but they can cause serious complications in patients with cardiovascular diseases, hypertension, high intracranial pressure, and cerebrovascular diseases. Moreover, due to the increased need for oxygen, they can lead to myocardial ischemia, myocardial infarction, cardiac arrhythmia, stroke, and increased morbidity and mortality [2-4].

A laryngoscope is a device for observing the larynx, the vocal cords, and the duct between them, which has various types. A laryngoscope consists of a long handle and a blade with a small light source at the top. The laryngoscope blade’s design has many forms, and the two most commonly used blades are the Macintosh and Miller blades, which are curved and straight, respectively. The Macintosh blade is easier to operate, while the Miller blade provides a better view of the vocal cords. The use of each of these blades depends on the habit and experience of anesthesiologists [5, 6].

In laryngoscopy with a Macintosh blade, the blade enters the vallecula, and the hyoepiglottic ligament is elevated without affecting the epiglottis, requiring the maximum extension of the neck while the tongue is pulled leftward and then subjected to a strong force pulling upward. However, the blade of the Miller laryngoscope is straight, and the epiglottis is lifted during laryngoscopy. In most cases, when the Macintosh laryngoscope cannot be used, the Miller laryngoscope is utilized because it requires less force and less neck extension. It is used in people with irregular teeth, especially those with missing right upper teeth [7-9]. The Miller blade is straight without any curvature, and due to the anatomy of the mouth and tongue and the large epiglottis in children, the Miller blade provides a clearer view of the larynx inlet [10].

Since children are sensitive and laryngoscopy is a difficult and painful action, the purpose of this study was to select a blade that would minimize injury to the patient. Therefore, this study was designed to compare two Macintosh and Miller blades for laryngoscopy in children less than 4-year-old.

## Material and Methods

A total of 72 children with a score of I and II, according to the American Society of Anesthesiologists (ASA) physical status classification, who were candidates for elective surgery with general anesthesia requiring intubation were enrolled in the study after obtaining written consent from their parents. Exclusion criteria were abnormal airway anatomy, history of active respiratory infections or cold in the past three weeks, history of chronic respiratory asthma, and allergies. The children were randomly divided into two groups. Laryngoscopy and intubation were performed with a Miller blade (group I) and with a Macintosh blade (group II). In both groups, children received oral premedication: midazolam 0.5 mg/kg and ketamine 2.5 mg/kg. Half an hour after sedation, EMLA anesthetic ointment was applied and venipuncture was performed with a 22-gauge angiocath. After entering the operating room, the standard monitoring system for pulse oximetry, electrocardiogram (ECG), and non-invasive blood pressure (NIBP) was installed.

Anesthesia was induced through inhalation of sevoflurane (8%) and 100% oxygen at a gas flow rate of 3 L/min. Laryngoscopy and endotracheal intubation were performed after appropriate anesthesia and injection of 5 mg/kg sodium thiopental and 1 μg/kg fentanyl. Anesthesia was continued with 2.5% isoflurane. During laryngoscopy, the epiglottis view was evaluated with the Miller blade in the first group and the Macintosh blade in the second group by an experienced anesthesiologist. The laryngoscopic view is classified through the Cormack-Lehane system:

• grade 1 - full view of glottis;
• grade 2 - only posterior extremity of glottis can be seen;
• grade 3 - only epiglottis can be seen;
• grade 4 - neither glottis nor epiglottis can be seen.

The larynx was observed by positioning the head and using external pressure on the larynx to obtain the best view. In this study, the time interval between laryngoscopy and endotracheal intubation, heart rate, systolic blood pressure, non-invasive arterial blood pressure, and hemoglobin saturation were measured and the number of endotracheal intubation attempts and complications were recorded for both groups.

## Results

In this study, 72 patients were divided into two equal groups of 36. The frequency distributions of gender, mean weight and mean age in the Miller and Macintosh groups are shown in Table 1. The results showed that there was no statistically significant difference between the two groups.

**Table 1:** Frequency of gender, weight, and age in the Miller and Macintosh groups.

Variable			Miller	Macintosh	p-value*
**Gender**	**Male**	**Number**	32	30	0.7
		**Percent**	88.9	83.33	
	**Female**	**Number**	4	6	
		**Percent**	11.1	16.67	
**Age Mean ± SD (months)**			16.75 ± 4.99	17.78 ± 9.19	0.5
**Weight Mean ± SD (gram)**			16988.57 ± 2431.99	16300 ± 4950.04	0.9

Note: * Differences between groups are statistically significant (р<0.05).

A comparison of the mean heart rate in the two groups showed that the p-value was 0.4 before laryngoscopy and 0.06 after laryngoscopy. In the Miller group, the heart rate was 131.4 before laryngoscopy and 137.4 after laryngoscopy (p-value=0.04). In the Macintosh group, the heart rate was 124.2 before laryngoscopy and 127.9 after laryngoscopy (p-value=0.047). The mean systolic and diastolic pressures before and after laryngoscopy are shown in Table 2.

**Table 2:** Mean systolic and diastolic pressures before and after laryngoscopy.

**Variable**	**Group**	**Mean ± SD**	p-value*
**Mean systolic/diastolic pressure before laryngoscopy**	Miller	131.92±17.12	0.4
	Macintosh	124.28±17.63	
**Mean systolic/diastolic pressure after laryngoscopy**	Miller	137.44±19.66	0.06
	Macintosh	127.97±18.9	

Note: * Differences between groups are statistically significant (р<0.05).

The mean oxygen saturation (SaO2) in minutes 1, 2, 3, 4, and 5 in the Miller and Macintosh group are shown in Table 3. According to the results, there was a significant difference between the two groups in terms of SaO2 only at minute 4, while the difference was not significant at other times.

**Table 3:** Mean SaO2 at different times in the Miller and Macintosh groups.

**Variable**	**Minute**	**Group**	**Mean ± SD**	p-value*
**SaO_2_**	1	Miller	99.81±0.47	0.5
		Macintosh	99.72±0.61	
	2	Miller	99.75±0.60	0.2
		Macintosh	99.56±0.69	
	3	Miller	97.44±1.23	0.6
		Macintosh	97.56±0.97	
	4	Miller	98.81±0.82	0.003
		Macintosh	99.33±0.63	
	5	Miller	99.64±0.49	0.3
		Macintosh	99.75±0.50	

Note: * Differences between groups are statistically significant (р<0.05).

The mean saturation decrease was 1.26 in the Miller group and 1.54 in the Macintosh group (p=0.3). Regarding the Cormack-Lehane classification system, 5 patients were classified as grade 1 (13.9%), 14 patients as grade 2 (38.9%), 15 patients as grade 3 (41.7%), and 2 patients as grade 4 (5.6%) in the Macintosh group. In contrast, 5 patients were classified as grade 1 (13.9%), 27 patients as grade 2 (75%), and 4 patients as grade 3 (11.1%) in the Miller group (p-value=0.004).

## Discussion

Management of airways during general anesthesia is the responsibility of anesthesiologists. Various methods, such as face mask, laryngeal mask, and endotracheal intubation, are used in this regard [11, 12]. Endotracheal intubation requires the use of a laryngoscope, which is available with different blades such as Macintosh and Miller [3, 13]. The aim of all of these devices is to provide a convenient view of the patient’s larynx for easy intubation [3, 14].

In a study conducted in India in 2014, Kundu et al. compared the Miller and Macintosh blades in children under 5 months under general anesthesia in terms of laryngoscopic view and ease and success of intubation. The authors found a similar view of the glottis in 43% of cases and a better view in 29% and 28% of cases when using the Miller and Macintosh blades, respectively. In addition, laryngoscopy was easily performed in 54% of cases, whereas it was performed difficultly in 27 children with both blades, in 15 children with the Miller blade, and in 13 children with the Macintosh blade [11].

There was a significant difference between the two groups in the mean heart rate at minute 3 (p-value=0.03), the mean systolic/diastolic pressure at all minutes, and the mean SaO2 at minute 4 (p-value=0.003). In a study by Bhardwaj conducted in 2013 to evaluate cervical spine motion with a Macintosh laryngoscope and a Trueview laryngoscope, the authors found that the latter offered a better laryngoscopic view of the glottis and less cervical spine motion [15].

In a study by Lu Yi that compared the Airtraq laryngoscope with the conventional Macintosh laryngoscope, the intubation time was reduced using the former (p<0.0001). Intubation was performed by both experienced and novice anesthesiologists (relative risk 1.25, p-value=0.07); therefore, the Airtraq laryngoscope facilitates and accelerates intubation [16].

Bein et al. compared the conventional curved blade laryngoscope and GlideScope laryngoscope in difficult airways and showed that the latter was significantly better than straight laryngoscope [17].

In a study from Singapore, Lye Sti compared endotracheal intubation with Macintosh laryngoscopy and indirect laryngoscopy and showed that the rate of successful intubation with indirect laryngoscopy (85%) was higher than Macintosh laryngoscopy (69%) [18].

In the present study, there was no significant difference between the two groups regarding the demographic variables (age, gender, and weight). Therefore, it seems that randomization and matching were appropriate, and there were no confounding factors in this regard in the study. Also, there was no significant difference in the mean blood pressure before and after laryngoscopy between the groups.

There was a significant increase in the heart rate before and after laryngoscopy due to stress in response to laryngoscopy. There was a statistically significant difference in the heart rate increase before and after laryngoscopy in the Miller group. Although changes in the heart rate were statistically significant, the increase in the heart rate was clinically less than 10% in the two groups and, therefore, did not require any special treatment.

There was no decrease in saturation in neither of the groups, and it seems that the laryngoscopy blades had no effect on the oxygen saturation of hemoglobin. The Cormack-Lehane classification of the laryngoscopic view was the most important variable in this study. In the Macintosh group, 5 patients were classified as grade 1, 14 patients as grade 2, 15 patients as grade 3, and 2 patients as grade 4. In contrast, in the Miller group, 5 patients were classified as grade 1, 27 patients as grade 2, and 4 patients as grade 3 (p-value=0.004).

## Conclusion

In general, the Cormack-Lehane system of the Miller blade was better than the Macintosh blade, giving a better laryngoscopic view. One finding of interest in this study is that older children had a better Cormack-Lehane grade. It seems that the laryngoscopic view improves as the age increases, and younger age is associated with a worse laryngoscopic view. This can be justified by the anatomy of the airway in children - their larynx is located anterior and superior, and their epiglottis is larger. As a result, there is a direct relationship between the improved laryngoscopic view and the Cormack-Lehane grade with age. However, there was no significant relationship between the two groups in terms of gender, and it seems that gender has no effect on the Cormack-Lehane grade.

## Conflict of Interest

The authors declare that there is no conflict of interest.

## References

[1] Magill I. (1930). Technique in endotracheal anaesthesia. British medical journal.

[2] Macintosh R. (1943). A new laryngoscope. Lancet.

[3] Arino J.J. (2003). Straight blades improve visualization of the larynx while curved blades increase ease of intubation: a comparison of the Macintosh, Miller, McCoy, Belscope and Lee-Fiberview blades. Canadian Journal of Anesthesia.

[4] Ray D. (2009). A comparison of McGrath and Macintosh laryngoscopes in novice users: a manikin study. Anaesthesia.

[5] Orebaugh S.L., Bigeleisen P.E. (2012). Atlas of airway management: techniques and tools.

[6] Benumof J. (1996). Conventional (laryngoscopic) orotracheal and nasotracheal intubation (single-lumen tube). Airway management. Principles and Practice.

[7] Rubio F.Q. (1995). Magill forceps: a vital forceps. Pediatric emergency care.

[8] Suzuki A. (2013). Use of a new curved forceps for McGrath MAC TM video laryngoscope to remove a foreign body causing airway obstruction. Saudi journal of anaesthesia.

[9] Soroudi A. (2007). Adult foreign body airway obstruction in the prehospital setting. Prehospital emergency care.

[10] Koomson A.K., Lavoie J. (2005). Broken fragment from a Magill forceps in the airway of a neonate. Canadian Journal of Anesthesia.

[11] Mattu A. (2012). Avoiding common errors in the emergency department.

[12] Asai T. (2003). Comparison of two Macintosh laryngoscope blades in 300 patients. British journal of anaesthesia.

[13] Smith C.E. (1999). Evaluation of tracheal intubation difficulty in patients with cervical spine immobilization: fiberoptic (WuScope) versus conventional laryngoscopy. Anesthesiology.

[14] Asai T. (2001). Evaluation of the disposable Vital View™ laryngoscope: apparatus. Anaesthesia.

[15] Bhardwaj N. (2013). Assessment of cervical spine movement during laryngoscopy with Macintosh and Truview laryngoscopes. Journal of anaesthesiology, clinical pharmacology.

[16] Lu Y., Jiang H., Zhu Y. (2011). Airtraq laryngoscope versus conventional Macintosh laryngoscope: a systematic review and meta-analysis. Anaesthesia.

[17] Serocki G. (2010). Management of the predicted difficult airway: a comparison of conventional blade laryngoscopy with video-assisted blade laryngoscopy and the GlideScope. European Journal of Anaesthesiology (EJA).

[18] Lye S.T. (2013). Comparison of results from novice and trained personnel using the Macintosh Laryngoscope, Pentax AWS®, C-MACTM, and Bonfils Intubation Fibrescope: a manikin study. Singap Med J.

